# Bistable states and frequency-dependent switching characteristics of the mesophases of a high dipole moment superparaelectric liquid crystal

**DOI:** 10.1039/d6ra05136g

**Published:** 2026-09-18

**Authors:** Mudit Sahai, Neelam Yadav, Yuri P. Panarin, O. E. Panarina, Wanhe Jiang, V. Manjuladevi, Georg H. Mehl, Jagdish K. Vij

**Affiliations:** a Department of Electronic and Electrical Engineering, Trinity College Dublin, The University of Dublin Dublin 2 Ireland JVIJ@tcd.ie; b Department of Physics, Birla Institute of Technology and Science, Pilani (BITS Pilani) Rajasthan 333031 India manjula@pilani.bits-pilani.ac.in; c Department of Physics, Motilal Nehru National Institute of Technology Allahabad Prayagraj 211004 India; d Department of Electrical and Electronic Engineering, TU Dublin Dublin 7 Ireland; e Department of Chemistry, University of Hull Hull HU6 7RX UK; f Shaanxi International Research Center for Soft Matter, State Key Laboratory for Mechanical Behavior of Materials, Xi'an Jiaotong University Xi'an 710049 P. R. China

## Abstract

The electro-optical (EO) switching properties of a rod-shaped liquid crystal (LC) molecule, WJ-16, which exhibits a molecular dipole moment of 10.8 D and superparaelectric (SPE) behaviour in its nematic and smectic-A (SmA) phases, are investigated. Similar measurements are also performed for the conventional paraelectric LCs, 8CB and 8OCB, and the results for the three LCs are compared to illustrate the differences between the SPE behaviour and the conventional paraelectric EO properties. The switching voltage (*V*_0.1_) required for the molecular reorientation from the planar to homeotropic state is used as the key parameter for these investigations. It is found to be highly frequency dependent for the nematic phase of WJ-16, whereas it remains almost frequency independent for both 8CB and 8OCB. The presence of nanoscale polar clusters in WJ-16 arises from the polarization–splay coupling, and these clusters facilitate the formation of clear bistable states in the SmA phase, as evidenced by a large optical memory effect. However, such effects are not observed in 8CB and 8OCB. The switching characteristics provide a clear distinction between the SPE material and the conventional paraelectric LCs. The observed characteristics indicate the potential of using WJ-16 in optical modulation and memory devices.

## Abstract 2

The electro-optical (EO) switching properties of a rod-shaped liquid crystal (LC) molecule, WJ-16, dipole moment of 10.8 D and superparaelectric (SPE) behaviour in its nematic and smectic-A (SmA) phases.

## Graphical abstract

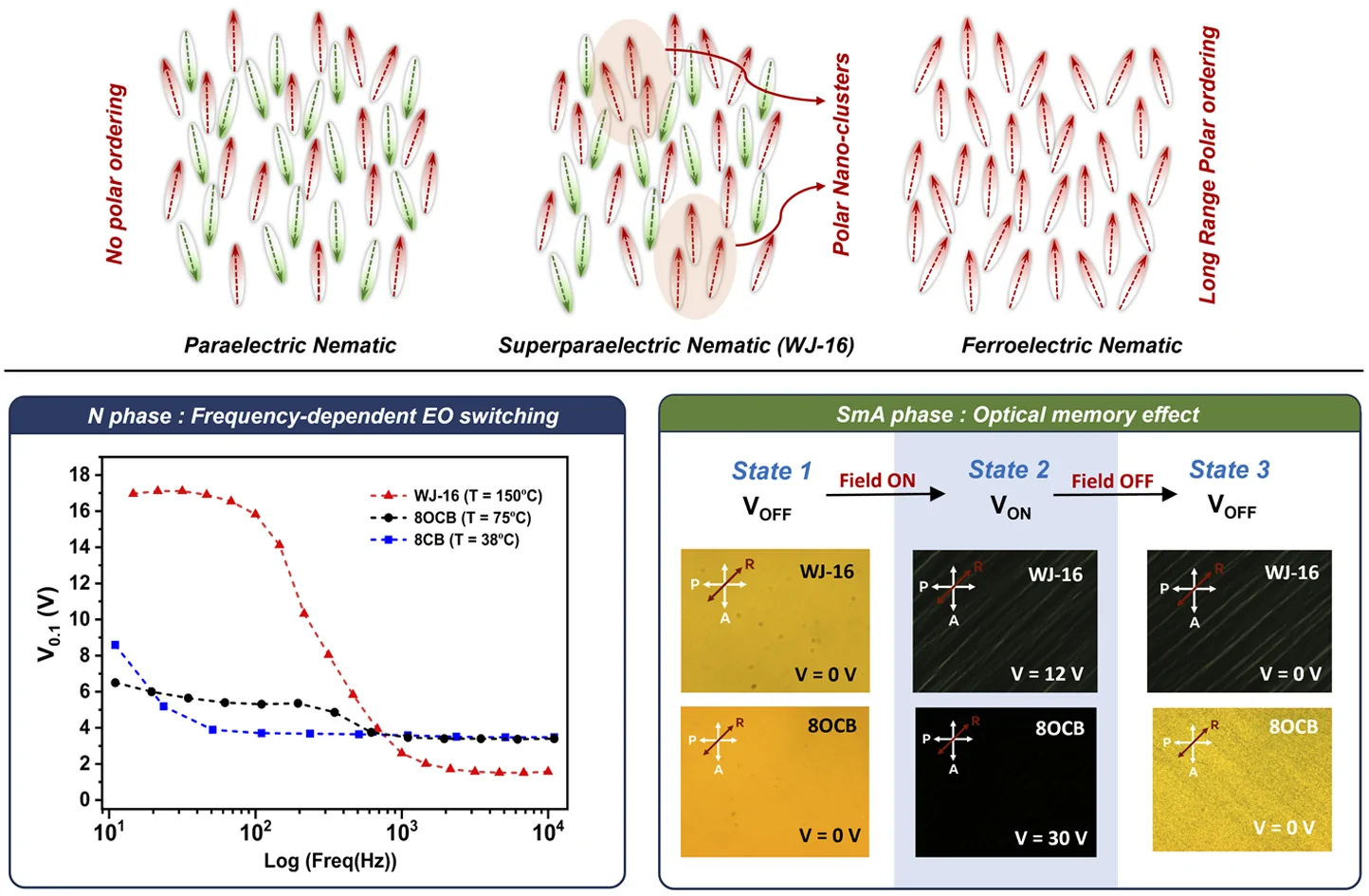

## Introduction

The discovery of the ferroelectric nematic phase in highly dipolar rod-like achiral DIO molecules, first reported by Nishikawa *et al.*^1^ in 2017, has rekindled the interest in the study of liquid crystalline (LC) molecules with large molecular dipole moments.^1,2^ This is a remarkable achievement since the initial theoretical prediction for such a polar nematic phase was made over a century ago by Born.^3^ These rod-shaped molecules that show ferroelectricity in the N phase have extremely large dipole moments (*µ*_D_ ≈ 10 D), but only some of them exhibit ferroelectric ordering in the nematic liquid crystal (LC) mesophase.^4–6^ The polar ordered state is associated with high spontaneous polarization (*P*_s_ ∼5 µC cm^−2^) and large dielectric permittivity (*ε* ∼10 000).^7–12^ High *ε* values for such materials are desirable characteristics for designing efficient charge storage devices, thus making them promising for the next generation of supercapacitors for use in high-power energy storage systems.^13–15^ Recently, we investigated two newly synthesized phenyl pyrimidine-based highly dipolar molecules (WJ-16 and WJ-18). These molecules exhibit colossal *ε* values in their LC mesophases but do not exhibit any macroscopic polar ordering.^16,17^ This superparaelectric (SPE) behavior arises since the field-induced polarization is linearly related to the applied field, this is large and is almost hysteresis free. These features are the first examples of the material characteristics observed in fluidic organic systems. Here, we report on the superparaelectric mesophase under the application of an external electric field to investigate and understand the electro-optical switching and relaxation dynamics of LC molecules in this phase. A detailed investigation is an important step towards progress in the novel field of superparaelectric fluids and to link research to potential electro-optical device technologies. This study also highlights the role played by the large dipole moment of molecules in achieving high dielectric permittivity in LC-based fluidic systems. In this study, the characteristics of WJ-16 are compared with those of the two conventional paraelectric LC mesophases: 4-*n*-octyl-4′-cyanobiphenyl (8CB) and 4-*n*-octyloxy-4′-cyanobiphenyl (8OCB) molecules.^18,19^

When an electric signal of sufficient magnitude is applied across a liquid crystal cell using a conventional paraelectric nematic (N) material in a planar alignment, the dipolar LC molecules easily reorient along the direction of the applied field since the N phase only has orientational ordering and no positional ordering. The LC molecules are free to rotate around their short axes, and by this mechanism, the constituent molecules are directed to align along the direction of the applied field. This reorientation of molecules from planar to homeotropic state is called the Fréedericksz transition.^20,21^ This transition is elastic in nature, which implies that when the applied field is removed, LC molecules relax (return) to their original positions. The Fréedericksz transition is of great applicational importance as it forms the basis of the LC nematic based display device industry. In contrast, when an electrical field is applied across the LC in its smectic phase, the presence of positional ordering prevents free rotations to occur and thus prevents the reorientation of the LC molecules in the direction of the field. We note that the inelastic transition in SmA requires a much stronger electric field and occurs first *via* the breaking up of the smectic layers of the medium. A complete molecular model for a possible bending of smectic layers under the application of an external electric or magnetic field was first given by Parodi *et al.*^22^ and Hareng *et al.*^23^ Following their works, transitions in the smectic A (SmA) phase have been explored for possible memory and smart window applications.^24–27^ It was observed, nevertheless, that under the application of a large AC voltage (*f* ∼1 kHz), the planar textures (state 1, transparent) of the SmA phase can be switched into a homeotropic state (state 2, opaque), which relaxes back into a scattering state (state 3, translucent) upon the removal of the field.^28^ The relaxation period (the memory effect) can range from a few seconds to a few days, depending on the material parameters of the LC. The generation of these tristable states makes SmA phase of a liquid crystalline material a good alternative to the polymer-dispersed liquid crystal (PDLC) for its potential use in memory and smart window applications.

In this work, the electro-optical (EO) switching properties of phenyl pyrimidine-based rod-like LC molecule (WJ-16) with a dipole moment of 10.8 D were studied. The purity of WJ-16 is investigated and is given in ref. 16, see https://doi.org/10.1039/d4tc03561e. This LC molecular system exhibits a high-temperature N phase, followed by the SmA phase (Table 1). Both LC phases exhibit characteristic SPE behavior. LC systems with high dielectric anisotropy (Δ*ε*) are expected to exhibit faster switching response with a lower switching voltage.^29^ Recent studies of LCs in the N_F_ phase have shown faster switching rates.^30^ However, the existence of macroscopic polar ordering in the N_F_ phase makes it difficult to achieve uniform alignment and even harder to homogenously switch and exhibit the Fréedericksz transition-like behavior under the action of an electric field.^31,32^ To explore the same for an SPE system, the switching characteristics of WJ-16 as a function of applied voltage, frequency and temperature were studied. We observed that in contrast to the ferroelectric nematic behaviour, WJ-16 shows uniform and complete switching in both the N and SmA phases. This study also highlights that, in the N phase, the switching voltage for WJ-16 is highly frequency-dependent. In contrast, both 8OCB and 8CB have almost constant switching voltages independent of the frequency of the field applied across the cell in the N phase. Furthermore, the switching voltage for WJ-16 was found to be much higher than of the conventional paraelectric nematic phase of LCs at lower frequencies (*f* < 500 Hz), while it was lower at higher frequencies. This frequency-controlled switching voltage is a characteristic of fundamental importance in fabrication of highly sensitive optical actuators and modulators. Furthermore, the WJ-16 LC system exhibited a complete optical memory effect for a small switching voltage in the SmA phase. In contrast, no such memory effect has been observed in paraelectric LCs. This feature of WJ-16 can be tailored to fabricate highly stable and energy-efficient bistable switching and memory devices. Results of the experiments highlight that WJ-16 differs from both the conventional paraelectric nematic and from recently discovered N_F_ LCs. The findings show that the SPE characteristic is not only useful for energy storage devices but has potential for electro-optical applications too.

**Table 1:** Phase transition temperatures of the three LC fluids of calamitic molecules observed by polarizing microscopy. The dipole moments of WJ-16, 8CB and 8OCB are taken from the literature

Molecule	Phases and transition temperatures	Dipole moment (*D*)
WJ-16	Cr 79 °C SmA 110.5 °C N 198 °C Iso	10.8
8OCB	Cr 35 °C SmA 65 °C N 80 °C Iso	7.3
8CB	Cr 21 °C SmA 33.5 °C N 40.5 °C Iso	6.2

## Experimental details

**Fig. 1:**
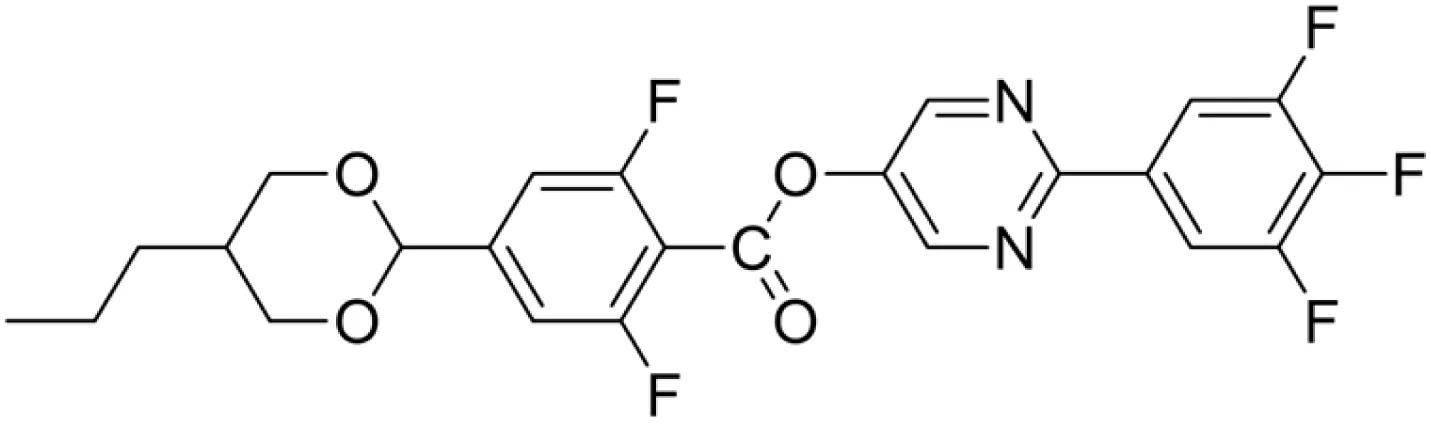
Chemical structure of the WJ-16 molecule.^16^

**Fig. 2:**
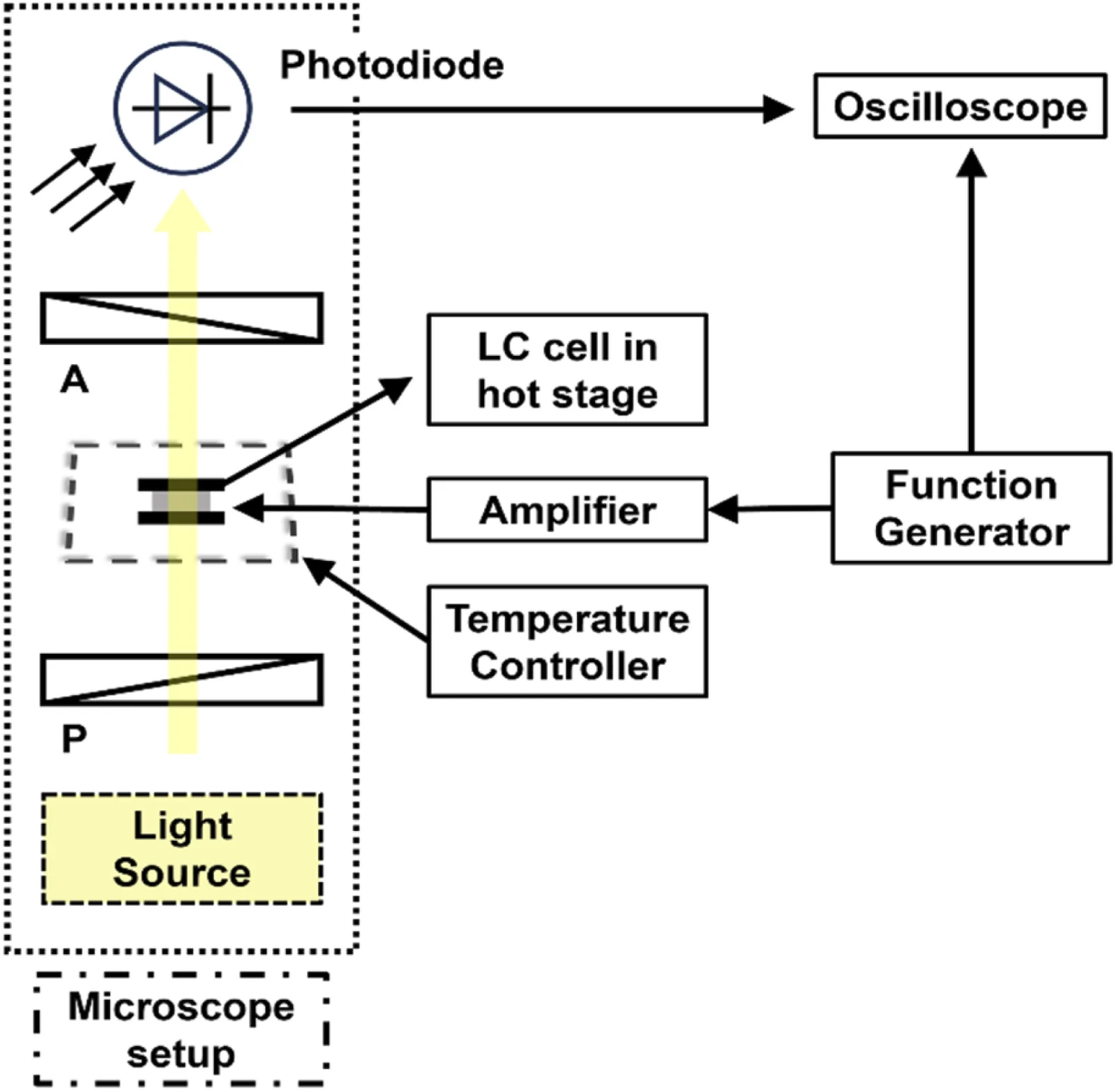
Electro-optical (EO) experimental setup using a polarizing optical microscope, where a liquid crystal (LC) cell is mounted in the hot stage. The polariser (P) and the analyser (A) of the microscope are crossed with each other.A photodiode is mounted in the camera port. The output is connected to a digital storage oscilloscope (DSO). A function generator connected to a voltage amplifier is used to trigger/apply an external voltage over the LC cell.

We studied fluids of three LC molecules having different dipole moments; among them, 8CB and 8OCB are the conventional LCs that exhibit paraelectric LC mesophases. Both LCs were procured from Sigma-Aldrich (purity >98%) and used without further purification. The third molecular fluid, WJ-16 (Fig. 1), was synthesized in the University of Hull and it exhibits interesting and unusual SPE characteristics in both the N and SmA LC phases.^16^ The phase transition temperatures of the three LC molecular fluids are given in Table 1. Electro-optic (EO) switching measurements were performed to determine differences in the molecular ordering between the conventional LC molecules and WJ-16, using the setup shown in Fig. 2. Commercial planar cells (EHC Co. Ltd, Japan) with a cell spacing of 2 µm were used for these measurements. The LC cells filled with the material were mounted in a hot stage, which, in turn, was fixed to the rotating stage between the crossed polarizers of the polarizing optical microscope (POM; Olympus BX52) setup. A digital camera was used to record the POM textures of the LC samples at different temperatures. For the switching experiments, the digital camera was replaced by a Hamamatsu C6386 photodiode receiver system, which measures the light intensity as a function of the applied voltage. Temperature of the hot stage is controlled using a Eurotherm 2604 temperature controller with a stability of ±0.01 °C. For POM studies, planar cells with the rubbing direction drawn parallel on two opposite substrates are kept at an angle of 45° from both the polarizer (P) and the analyser (A) positions. These are in crossed positions. The electric field is applied across the cell from an Agilent 33120A signal generator and is amplified by a high-voltage amplifier (TReK PZD700). The optical switching characteristics were studied as a function of temperature and frequency of the applied voltage for the three LC systems. Each set of measurements was carried out under a cooling/frequency cycle. For example, during a cooling cycle, measurements began when the LC sample is in the N phase: a voltage at a fixed frequency (1 kHz) is applied across the cell, and its magnitude is increased in regular steps, with the transmitted intensity recorded at each step. When switching from the planar to the homeotropic state finally occurs, the transmitted intensity goes from bright to dark, *i.e.* from the maximum to the minimum. Thereafter, magnitude of the applied voltage is decreased back to zero; thus, the initial voltage step is retraced, and the planar state recovered. The temperature is then reduced. Similar cyclic measurements were repeated as a function of temperature until we reached deep into the SmA phase of each of the three LC samples. The measurements were also performed as a function of frequency at a fixed value of the applied voltage in the N phase. The recorded results showed characteristic differences between conventional LCs and WJ-16.

## Results and discussions

### EO switching studies in the N phase

**Fig. 3:**
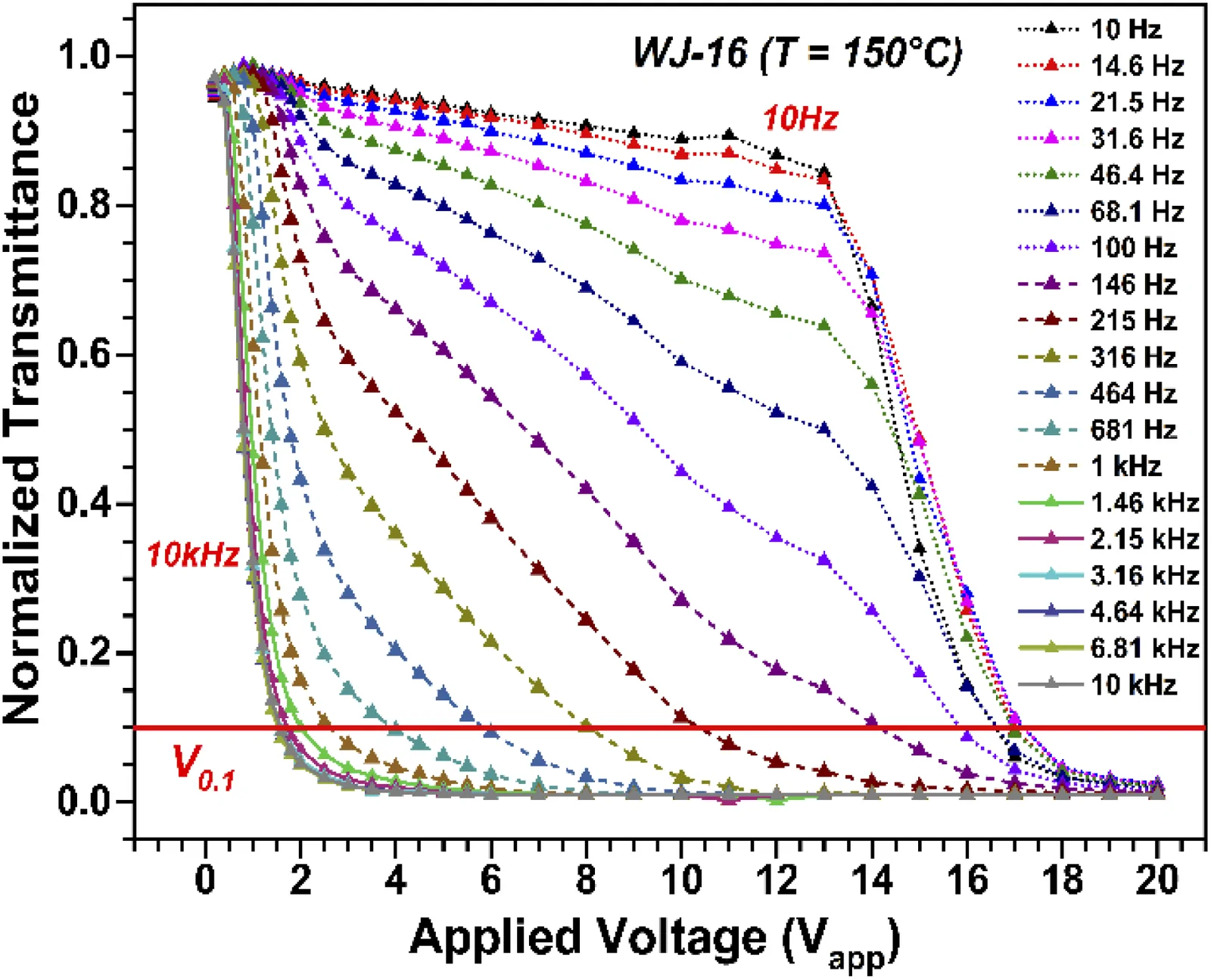
Evolution of the normalized transmittance in WJ-16 at *T* = 150 °C (N) as a function of the magnitude of applied AC voltage (*f* = 1 kHz, square waveform) at various frequencies.

**Fig. 4:**
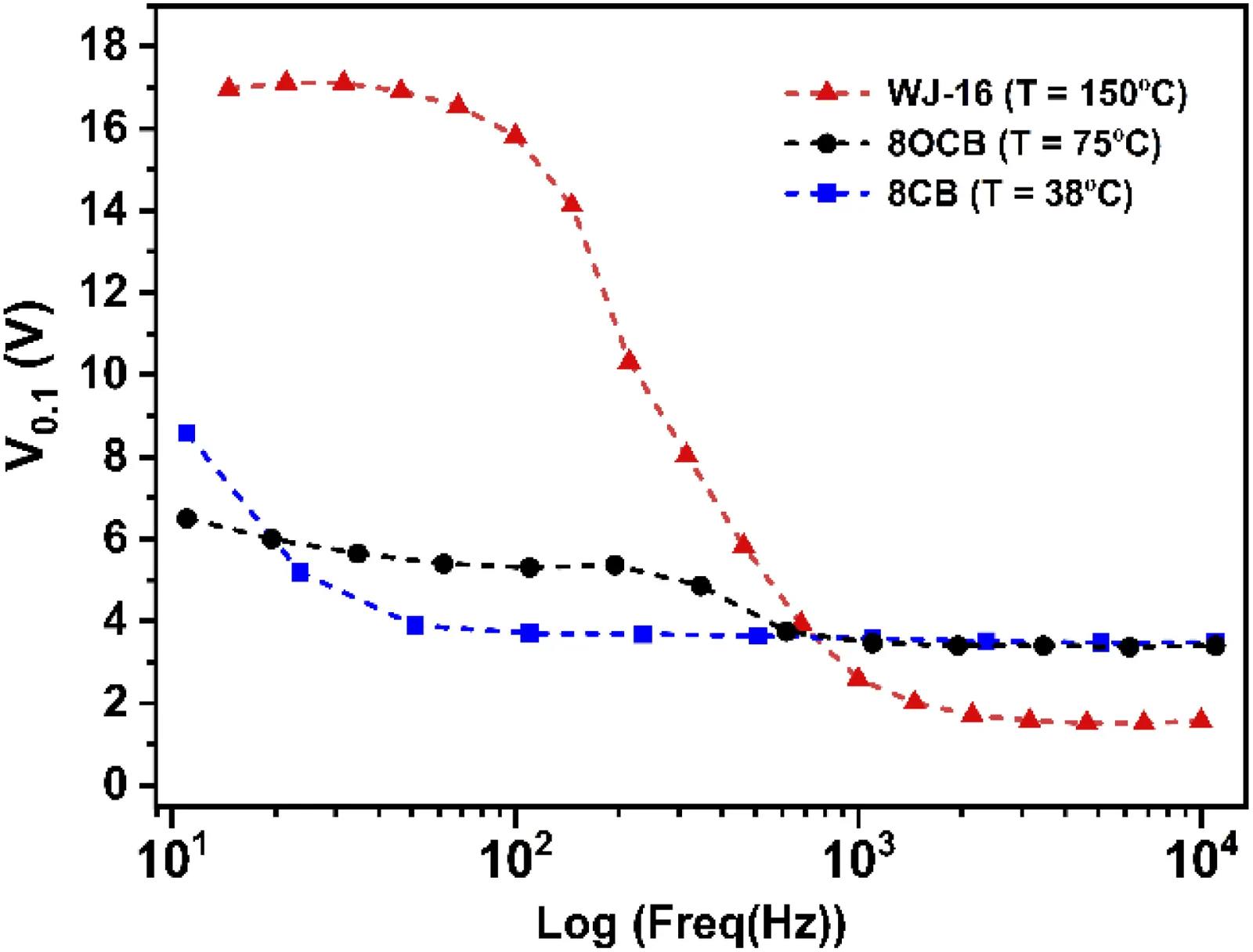
Variation in *V*_0.1_ (here, *V*_0.1_ refers to the voltage required to decrease the transmittance to 10% of its initial value at the start of the voltage cycle) for the WJ-16, 8OCB, and 8CB molecular systems as a function of the frequency of the applied voltage in the N phase.

The EO switching in the N phase is generally ‘a frequency-independent phenomenon’ that exhibits collective reorientation of the LC molecules under the applied electric field (SI Fig. S1 and S2). However, WJ-16 exhibited a variation in the normalized transmittance as a function of frequency of the applied voltage (*V*_app_) at *T* = 150 °C (Fig. 3). To clearly study this behaviour, we traced *V*_0.1_ from this figure. Here, *V*_0.1_ refers to the voltage required to reduce the transmittance of the initial bright-state value to its 10%. Similar measurements were performed for 8CB and 8OCB. *V*_0.1_ as a function of frequency was determined from the data, and the values thus obtained are plotted in Fig. 4. For WJ-16, *V*_0.1_ shows a strong frequency dependence, while 8CB and 8OCB exhibit variations at low frequencies (*f* < 50 Hz) only. Such low frequency variations in the paraelectric LCs possibly occur from the ionic impurities in the N phase, and the charges from the impurities shield the LC molecules from the signal of the applied voltage.^33,34^ However, for WJ-16, the dependence of *V*_0.1_ on frequency is much stronger even at higher frequencies. This is explained by the presence of clusters, which at the nanoscale level contribute to the low-frequency relaxation process and thus to the component of the permittivity parallel to the director. It has been found that the applied voltage is screened by the large permittivity of the system; hence a larger voltage is needed for electro-optical switching to occur. For a paraelectric nematic, the saturation voltage (*V*_sat_) for the Fréedericksz transition is given by ^20^^,^^21^1
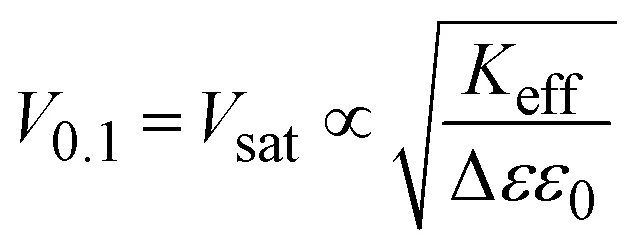
where Δ*ε* = (*ε*_∥_ − *ε*_⊥_) is the dielectric anisotropy, *K*_eff_ is the effective elastic constant of the LC, and *ε*_0_ is the permittivity of free space. In the conventional paraelectric nematics, 8CB or 8OCB, Δ*ε* is essentially frequency independent in the studied frequency range, where no additional internal degrees of freedom are involved. This means that *ε*_∥_ and *ε*_⊥_ behave similarly at lower frequencies. *V*_sat_ therefore remains nearly constant with frequency, apart from a slight increase observed at lower frequencies. This arises from ions screening the nanoscale sized polar clusters. A presence of these polar domains in the SPE phase introduces an additional dynamic variable: the field-induced polarization P. This polarization is coupled to the local director, and the field-induced splay deformation perturbs the polarization distribution. This distortion contributes to the total free energy through electrostatic and polarization–director coupling terms, thereby increasing the energetic cost of the splay deformations. This polarization–splay coupling increases the effective splay constant, which resists the director deformations, and thus, higher *V*_0.1_ values are obtained for WJ-16 at lower frequencies. This polarization-splay coupling increases the effective splay constant, but the twist and bend elastic constants decrease, as recently reported for the N_F_ phase.^35,36^ This happens because at lower frequencies, the polarization (or polar clusters) can follow the field, thus maximizing elastic stiffening. This results in higher *V*_0.1_ values when compared to conventional nematics. At higher frequencies (*f* > 1 kHz), response of polarization to the field diminishes and its coupling to director distortions weakens. This reduces the effective splay constant, while the dielectric anisotropy, Δ*ε* = (*ε*_∥_ − *ε*_⊥_) ∼ 10^3^, in which *ε*_∥_ and *ε*_⊥_ are the two components of permittivity, exhibits strong dispersion in the 100 Hz–1 kHz range.^17^ Thus, we observe lower *V*_0.1_ values at higher frequencies for WJ-16. However, these components are almost frequency independent for cyanobiphenyls up to a frequency of 1 MHz.^37^ The existence of this frequency-dependent *V*_0.1_ in WJ-16 opens up new possibility of applications of this highly dipolar LC molecular system in sensors and actuators. Furthermore, lower *V*_0.1_ values above 1 kHz for WJ-16 enables it to be used in higher energy-efficient switching devices.

### Evolution of the switching voltage across the N-SmA transition

The observed POM textures for both WJ-16 and 8OCB in the SmA phase under the effect of an applied voltage are shown in SI, Fig. S3. Here, both samples were kept at 5 °C below the N-SmA transition temperatures. These are expressed in terms of the normalized transition temperature, (*T* − *T*_NA_)/*T*_NA_, where *T*_NA_ is the N to SmA transition temperature in the degree centigrade (°C). This approach is useful because translational ordering of the LC molecules in the SmA phase compared to in the N phase prevents uniform EO switching. Upon the application of *V*_app_, striated texture appears, which grows as magnitude of the voltage increases, and finally leads to the formation of a quasi-homeotropic state (SI Fig. S3). Similar textures were observed in all three LC molecular systems. Although the observed textures and their evolution with voltage are similar, magnitude of the *V*_app_ required for these transitions varies widely across the studied LCs. WJ-16 requires a 50% to 60% lower *V*_app_ at a similar normalized temperature to switch from the planar to homeotropic state compared to that required for 8OCB and 8CB.

**Fig. 5:**
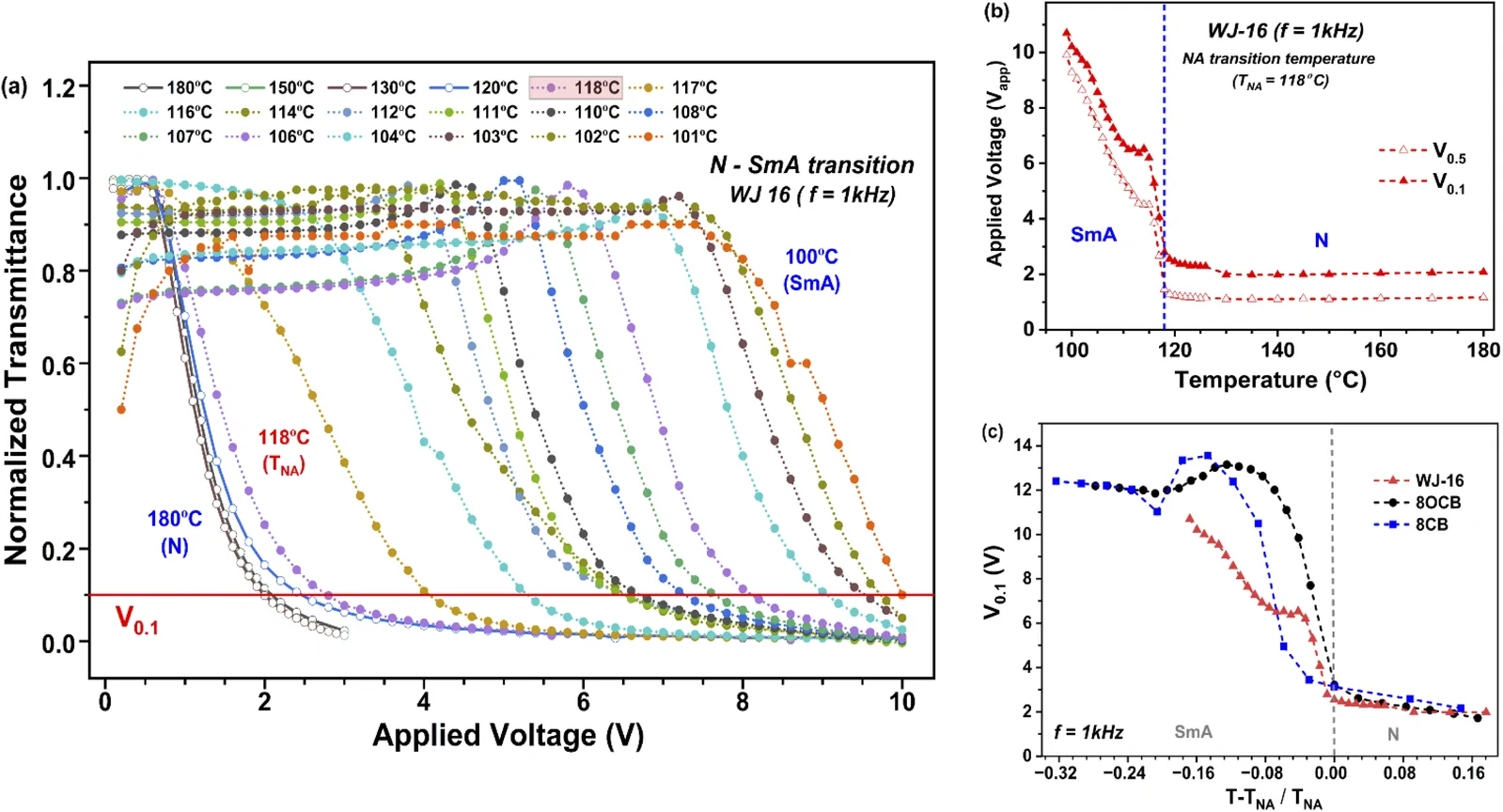
(a)Variation in the normalized transmittance under the application of an AC voltage (*f* = 1 kHz, square waveform) for WJ-16 as a function of temperature. Here, the solid lines indicate the nematic phase, while the broken lines signify the SmA phase. The N to SmA transition temperature (118 °C) is shown as *T*_NA_. (b) Variation in *V*_0.5_ and *V*_0.1_ across the N-SmA phase transition temperature for the WJ-16 molecule filled in a planar cell of 2 µm thickness. Here, *V*_0.5_ refers to the voltage required to decrease the transmittance to 50% of the initial value at the start of the cycle. Similarly, *V*_0.1_ refers to the voltage needed to decrease the transmittance to 10% of the initial value at the start of the cycle. (c) Variation in the *V*_0.1_ value of the WJ-16, 8OCB, and 8CB molecules as a function of the normalized transition temperature, (*T* − *T*_NA_)/*T*_NA_, across the N-SmA transition temperature.

In addition, by studying the evolution of *V*_0.1_ with temperature, we can investigate nature of the N-SmA transition in WJ-16 and compare it with a similar transition observed in 8CB and 8OCB sample. Previously, some works have used the EO switching phenomena for the elastic (N phase) and the non-elastic deformations (SmA phase) in characterizing the behaviour of the N-SmA transition in LC systems.^38–40^ Most of these efforts were concerned with exploring the reorientation and relaxation mechanism of LC molecules in the SmA phase in different cell geometries. However, we used a similar technique to examine the effect of the dipole moment and dielectric permittivity on the molecular ordering and phase transition properties across the N-SmA transition. Fig. 5a shows the variation in the normalized transmittance as a function of the applied voltage (*f* = 1 kHz) at different temperatures for WJ-16. From this plot, variations of *V*_0.5_ and *V*_0.1_ as a function of temperature were noted, and these are plotted in Fig. 5b. Here, *V*_0.5_ refers to the voltage required to decrease the transmittance to 50% of the initial bright state value. Similarly, *V*_0.1_ refers to the voltage needed to decrease the transmittance to 10% of the initial value. Similar measurements were carried out for 8CB and 8OCB using commercial planar cells of the same thickness as for WJ-16 (SI Fig. S4 and S5), and the observed results for all the three molecules are combined and plotted in Fig. 5c. This plot shows some key observations:

• In the N phase, where the conventional EO switching occurs, the three LC samples show similar *V*_0.1_ values for *f* = 1 kHz. Also, a complete reversible reorientation is observed upon the removal of *V*_app_.

• As the N-SmA transition temperature is reached, we observe a steep rise in *V*_0.1_ values due to an increase in molecular ordering and because of the formation of the smectic layers. Parallelly, the switching voltage (*V*_0.1_) and its temperature dependence is studied, with WJ-16 requiring almost 50% lower *V*_0.1_ values than for 8CB and 8OCB. Generally, the planar-to-homeotropic reorientation in the SmA phase is described by the Parodi model,^22^ which states that the field-induced transition initially occurs as reorientation of the molecules in the central part of the cell. The *V*_0.1_ (or *V*_sat_) value for such a transition should follow eqn (1). However, this does not happen, mainly because of the strong divergence of the bend elastic constant, *K*_33_, upon approaching the SmA phase from the N phase and due to the large layer compression modulus, *B*.^40,41^ Both these features make the conventional Fréedericksz transition unobservable in the SmA phase. In contrast, as magnitude of the applied voltage increases, the uniform texture of SmA starts breaking up because the striation gradually travels along the smectic layers and extends throughout the smectic phase (SI Fig. S3C and G). If the voltage is increased further, the sample turns completely into a quasi-homeotropic state, which is observed as dark optical texture (SI Fig. S3D and H). The saturation voltage for the striation follows the. However, in SmA phase, the amplitude of the distortion, *θ*_m_, of the layer normal from the equilibrium position close to the saturation voltage (or electric field) is given by ^40–42^2
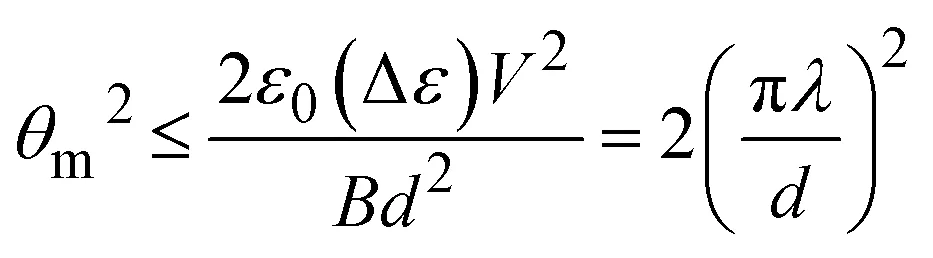
where *λ* is the smectic characteristic length, *λ* = (*K*_11_/*B*)^1/2^, and is generally of the order of a few smectic layer thicknesses. Δ*ε* is the dielectric anisotropy of the molecular system, *K*_11_ is the splay elastic constant, *d* is the cell thickness, *ε*_0_ is the permittivity of free space and *V* is the applied voltage. Using eqn (2), differences in *V*_0.1_ just below *T*_NA_ among the three molecules arise from their different Δ*ε* values. Furthermore, experimental data were recorded at *f* = 1 kHz. Hence, the higher dipole moment and higher dielectric permittivity of WJ-16 than for 8OCB and 8CB result in a much larger Δ*ε*, which in turn decreases *V*_sat_ (Fig. 5c). This SPE nature of the material in the filled cell leads to a formation of short-ranged polar clusters in WJ-16, which reorient and relax collectively. These aid in reducing *V*_0.1_ values.

• In both 8CB and 8OCB, we observe saturation in *V*_0.1_ values as temperature decreases further below the N-SmA transition temperature. In contrast, WJ-16 shows a completely different behaviour with a continuous increase in *V*_0.1_ as temperature decreases. One possible reason for this result could be a higher viscosity of WJ-16 arising from nanoscale sized clusters, leading to higher dielectric permittivity values.^43^

### Existence of a large optical memory effect (bistable states)

**Fig. 6:**
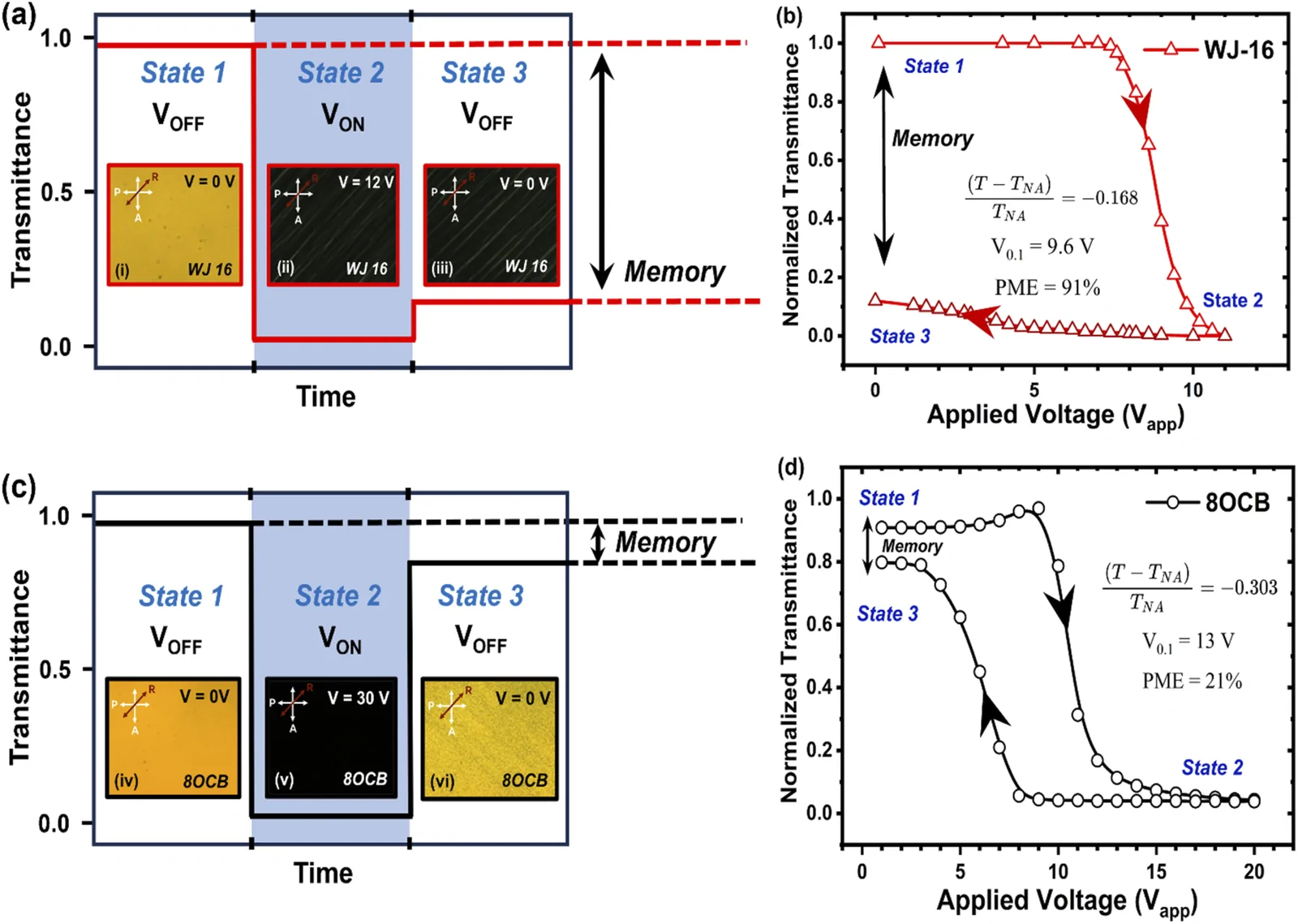
(a and c) are schematic that show variation in the normalized transmittance under the application of an AC voltage for *f* = 1 kHz. The inset shows the recorded POM textures at the given voltage ON and OFF states. Both the plots clearly show the strong optical memory effect exhibited by WJ-16, while no similar effect was observed in 8OCB. (b and d) Experimentally observed variation in the normalized transmittance as a function of applied cyclic AC voltage at a fixed frequency of 1 kHz. These plots further show the existence of a strong optical memory effect with large PME and small *V*_0.1_ value in the SPE-exhibiting WJ-16 sample as compared to the paraelectric 8OCB sample.

The SmA phase has been explored for bistable devices due to its inelastic EO switching. However, low efficiency and requirement for a higher switching voltage of the cell are the two major drawbacks of SmA-based devices.^44,45^ In the present work, WJ-16 was studied for the existence of memory effect, and the results were compared with 8OCB. Planar cells having cell spacings of 2 µm were used for these measurements. The observed results of transmittance are plotted as a function of a normalized transition temperature (*T* − *T*_NA_)/*T*_NA_. As shown in Fig. 6, when both WJ-16 and 8OCB are brought to 20 °C below their respective N-SmA transition temperatures ((*T* − *T*_NA_)/*T*_NA_ = −0.168 for WJ-16 and (*T* − *T*_NA_)/*T*_NA_ = −0.303 for 8OCB), under the application of an external AC voltage for *f* = 1 kHz, a transition from the planar to homeotropic state is observed in both samples. However, when the applied voltage is removed, 8OCB relaxes back to quasi-planar scattering state (Fig. 6c and d), while WJ-16 retains the homeotropic state almost perfectly, and molecules do not return to their initial planar states (Fig. 6a and b). This complete optical memory effect has not previously been observed in a conventional paraelectric SmA phase. This effect can clearly be observed during the evolution of the optical textures (SI Fig. S6) and from dependence of the normalized transmittance as a function of the cyclic voltage (Fig. 6c and d).

The observed memory effect is even more surprising given a lower thickness of the cell used in present study. In thinner cells, effect of the surface anchoring is greater, forcing LC molecules to return to their original planar states when voltage across the cell is removed. This is the reason behind the observation of an almost complete return of 8OCB and 8CB molecules to the original focal conic planar textures once *V*_app_ is removed (SI Fig. S7). In contrast, WJ-16 molecules retain a perfect homeotropic state in the absence of *V*_app_. This could be due to short-range polar clusters present in WJ-16, which reorient together upon the application of an external voltage and are stabilized in homeotropic state. We observed that WJ-16 favours homeotropic ordering even when filled in an uncoated cell.^16^ Thus, we can assume that when the temperature is lowered well below the N-SmA transition temperature, the polar clusters in WJ-16, along with the material's increased viscosity, do not overcome the surface anchoring strength of the homeotropic state. Hence, the homeotropic state is retained. To better quantify the advantage of WJ-16 as an optical memory system in comparison to the other LC-based fluidic systems, the permanent memory effect (PME) in % is calculated using the following equation:^46–48^3
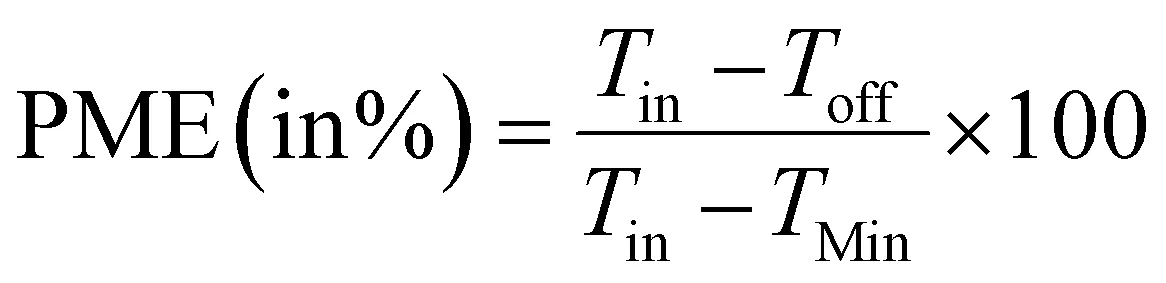
where *T*_in_ is the transmittance of the initial bright planar state, *T*_Min_ is the minimum transmittance in the homeotropic state, and *T*_off_ is the transmittance of the cell after the voltage is removed. PME measures the device efficiency for retaining the molecular ordering after *V*_app_ is removed. Over the last few decades, various LC-based devices have been studied for potential optical memory applications. In comparison, WJ-16 shows better PME (∼92%), a lower saturation voltage (∼12 V), and a higher stability (Fig. 6b).

**Fig. 7:**
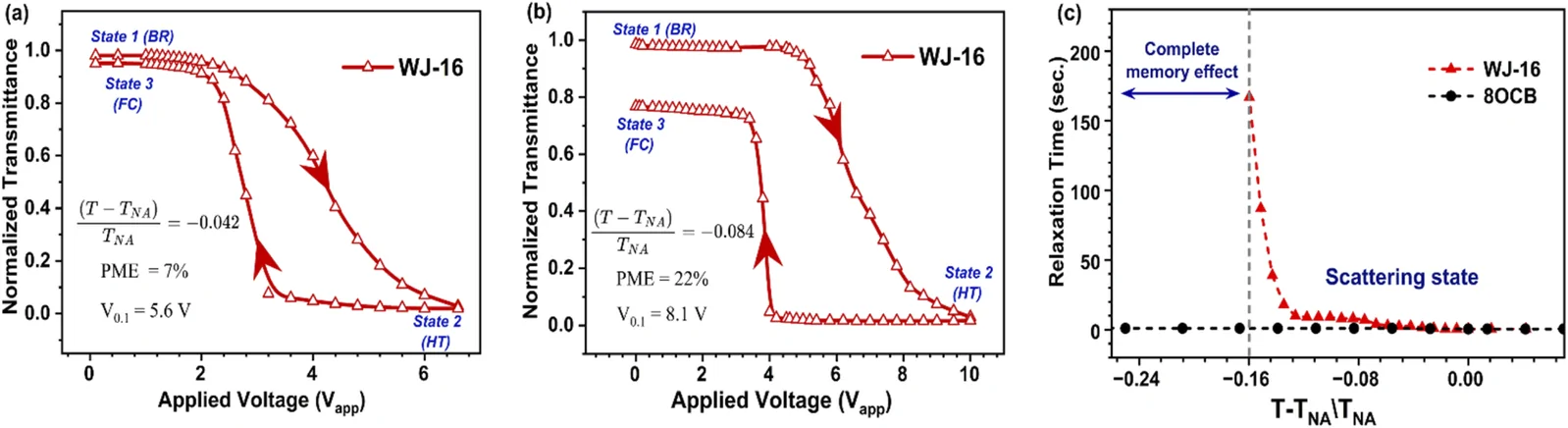
(a and b) Evolution of the optical memory effect observed in the WJ-16 molecule at different normalized transition temperatures. Here, BR refers to the initial bright state, HT represents the voltage-induced homeotropic state, and FC refers to the scattering focal conic state. (c) Variation in the relaxation time (time taken by the LC molecules to return to their original planar configuration after *V*_app_ is removed) for WJ-16 and 8OCB as a function of normalized transition temperature, (*T* − *T*_NA_)/*T*_NA_.

Apart from the memory effect, WJ-16 exhibits another important physical property below the N-SmA transition temperature, which could be used for controlled dynamic scattering applications such as smart windows and optical shutters. When the normalized transition temperature, (*T* − *T*_NA_)/*T*_NA_ of WJ-16 is between 0 and −0.16, it shows a reversible memory effect between the scattering focal conic (FC) state and the homeotropic (HT) state. This effect is similar to that observed in 8OCB (SI Fig. S7(D and E)), though WJ-16 shows better voltage responsivity and requires a 50% lower saturation voltage for switching between these two states (Fig. 7a and b). Thus, near *T*_NA_, the memory effect is not dominant and WJ-16 shows fast relaxation to the scattering state when the *V*_app_ is removed. Hence, we obtain a tristable system and a reversible memory effect. However, when the temperature is reduced further, we get a permanent or complete memory effect and a bistable system. Both characteristics are controlled by temperature. The same effect is verified and plotted in Fig. 7c, where the relaxation time for WJ-16 to return from the homeotropic state to the focal conic texture after *V*_app_ removal is shown as a function of the normalized transition temperature, (*T* − *T*_NA_)/*T*_NA_. The relaxation time was determined directly from the POM videos of the WJ-16 and 8OCB LC samples recorded immediately after the removal of the applied electric field. It was defined as the time required for the voltage-induced homeotropic state to completely relax to the scattering focal conic state. Representative POM videos for the relaxation process at different temperatures are given in the SI (SIMov1–SIMov4). Fig. 7c clearly shows that a complete memory effect is observed once the WJ-16 sample is cooled 20 °C below the N-SmA transition temperature. Above this temperature, WJ-16 forms a tristable scattering state on the removal of *V*_app_. However, no similar effects were observed for the paraelectric 8OCB sample.

## Conclusion

Here, we investigate the EO switching characteristic of a highly dipolar rod-shaped LC molecular fluid, WJ-16, which exhibits superparaelectric (SPE) nematic and smectic A phases. Similar measurements are also carried out for the conventional paraelectric 8CB and 8OCB LC systems. Results of the three LC systems are compared with each other. The saturation voltage (*V*_sat_ or *V*_0.1_) required for the reconfiguration of molecular orientation from planar to homeotropic ordering is used as the investigative parameter. The variation of *V*_sat_ is explored as a function of the frequency of the applied voltage signal and temperature of the LC sample. We found that *V*_sat_ for WJ-16 is highly frequency dependent in the N phase, while *V*_sat_ is almost frequency independent for both 8CB and 8OCB. This is attributed to a higher dipole moment molecular system and to the polar domains which account for the SPE behaviour of WJ-16. At lower frequencies, the polar domains in WJ-16 can follow the applied field variations, and these contribute significantly to the restoring torque which result in higher *V*_sat_ values. However, as frequency increases, their response becomes progressively weaker. This reduces the restoring torque and consequently the *V*_sat_ values are reduced. Furthermore, a variation in the *V*_sat_ as a function of temperature across the N-SmA transition for a fixed frequency (*f* = 1 kHz) is studied. Here, WJ-16 exhibits much lower *V*_sat_ values compared to those for 8CB/8OCB. This suggests that close to the N–SmA transition, polar domains assist in the field-induced director reorientation. The coupling of these domains with the local director provides an additional field-induced torque, thereby facilitating the planar-to-homeotropic transition at lower applied voltages compared to conventional paraelectric nematics studied here. The SPE behaviour also assists in retaining an almost complete memory state well within the smectic phase even after the applied electric field is removed. This optical memory of WJ-16 has a large permanent memory effect (PME) with a lower *V*_sat_ requirement, and thus, WJ-16 can potentially be used in bistable and optical memory devices.

## Author contributions

MS, NY, YP and JKV conceptualized the project. MS and YP performed the experiments. VM, WJ, GM and JKV assisted in data analysis. OEP assisted in the laboratory work and data analysis. VM and JKV did the overall project administration, and with JKV, MS and GHM reviewed, edited, and finalized the manuscript. All authors have approved the final version of the manuscript.

## Conflicts of interest

There are no conflicts to declare.

## Supplementary Material

RA-OLF-D6RA05136G-s001

RA-OLF-D6RA05136G-s002

RA-OLF-D6RA05136G-s003

RA-OLF-D6RA05136G-s004

RA-OLF-D6RA05136G-s005

## Data Availability

Data will be available from the corresponding authors on request. Supplementary information (SI) is available. See DOI: https://doi.org/10.1039/d6ra05136g.
