# Supplementary material for: Bistable states and frequency-dependent switching characteristics of the mesophases of a high dipole moment superparaelectric liquid crystal
Source: RSC Adv. 2026 Sep 18;16(44):55723–31. doi: 10.1039/d6ra05136g (PMC13587355; doi:10.1039/d6ra05136g)
Supplement: Supplementary file 1 [file RA-016-D6RA05136G-s001.pdf]

Supplementary Information (SI) for RSC Advances.
This journal is © The Royal Society of Chemistry 2026

Supplementary Information

Bistable states and the frequency dependent switching characteristics of the mesophases of a high dipole moment superparaelectric liquid crystal

Mudit Sahai,[a,b] Neelam Yadav,[a,c] Yuri P. Panarin,[a,d] O. E. Panarina,[a] Wanhe Jiang,[e] V. Manjuladevi,*[b] Georg H. Mehl,[e,f] Jagdish K. Vij*[a]

[a]Department of Electronic and Electrical Engineering, Trinity College Dublin, University of Dublin, Dublin 2, Ireland
[b]Department of Physics, Birla Institute of Technology and Science, Pilani (BITS Pilani), India
[c]Department of Physics, Motilal Nehru National Institute of Technology Allahabad, Prayagraj, 211004, India
[d]Department of Electrical and Electronic Engineering, TU Dublin, Dublin 7, Ireland
[e]Department of Chemistry, University of Hull, Hull HU6 7RX, UK
[f]Shaanxi International Research Center for Soft Matter, State Key Laboratory for Mechanical Behavior of Materials, Xi'an Jiaotong University, Xi'an 710049, P. R. China

*Corresponding authors: JVIJ@tcd.ie, manjula@pilani.bits-pilani.ac.in

1. EO switching studies in N phase in conventional paraelectric LCs:

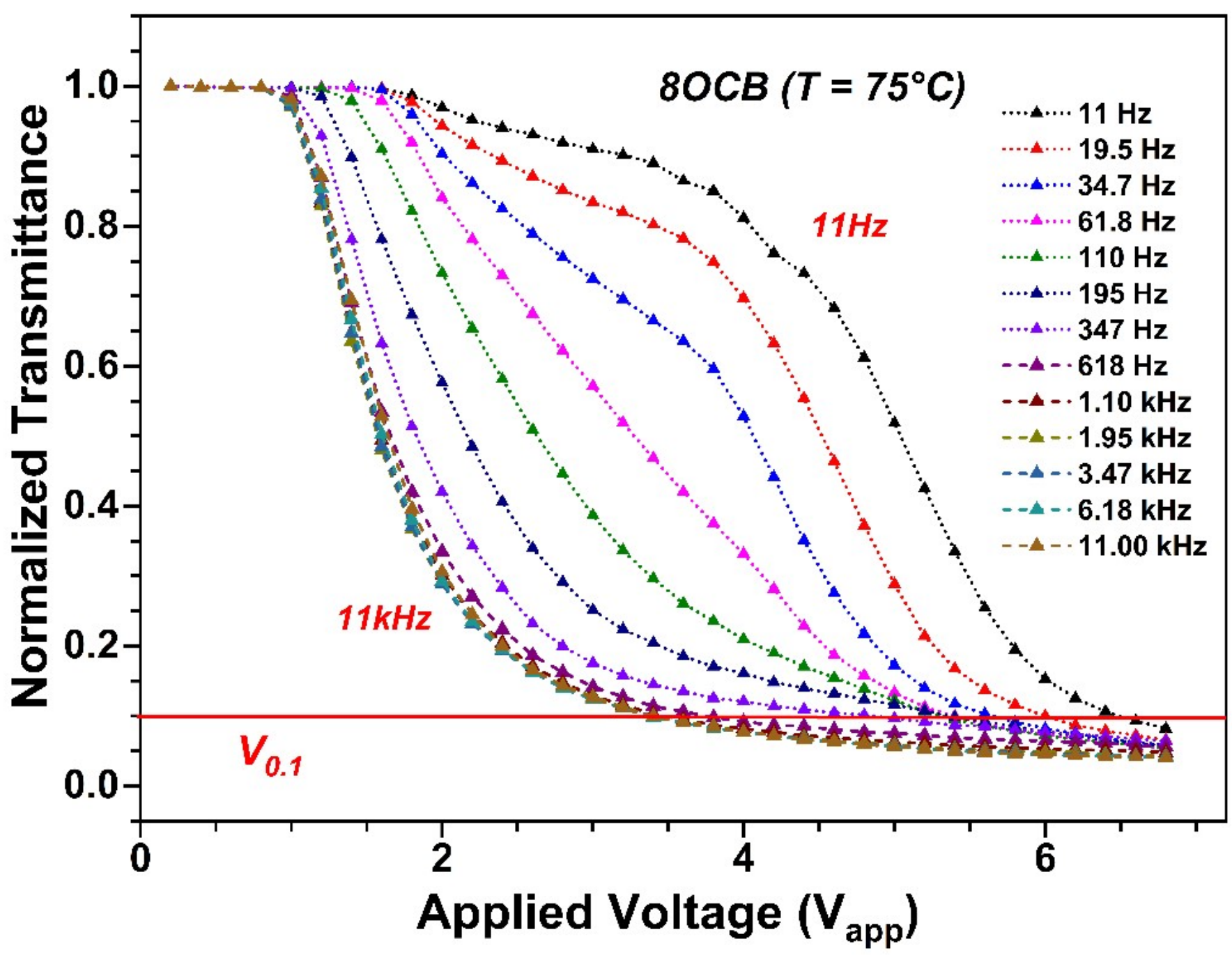

Figure S1: Evolution of normalized transmittance in 8OCB at T = 75°C (N) as a function of magnitude and frequency of the applied AC voltage.

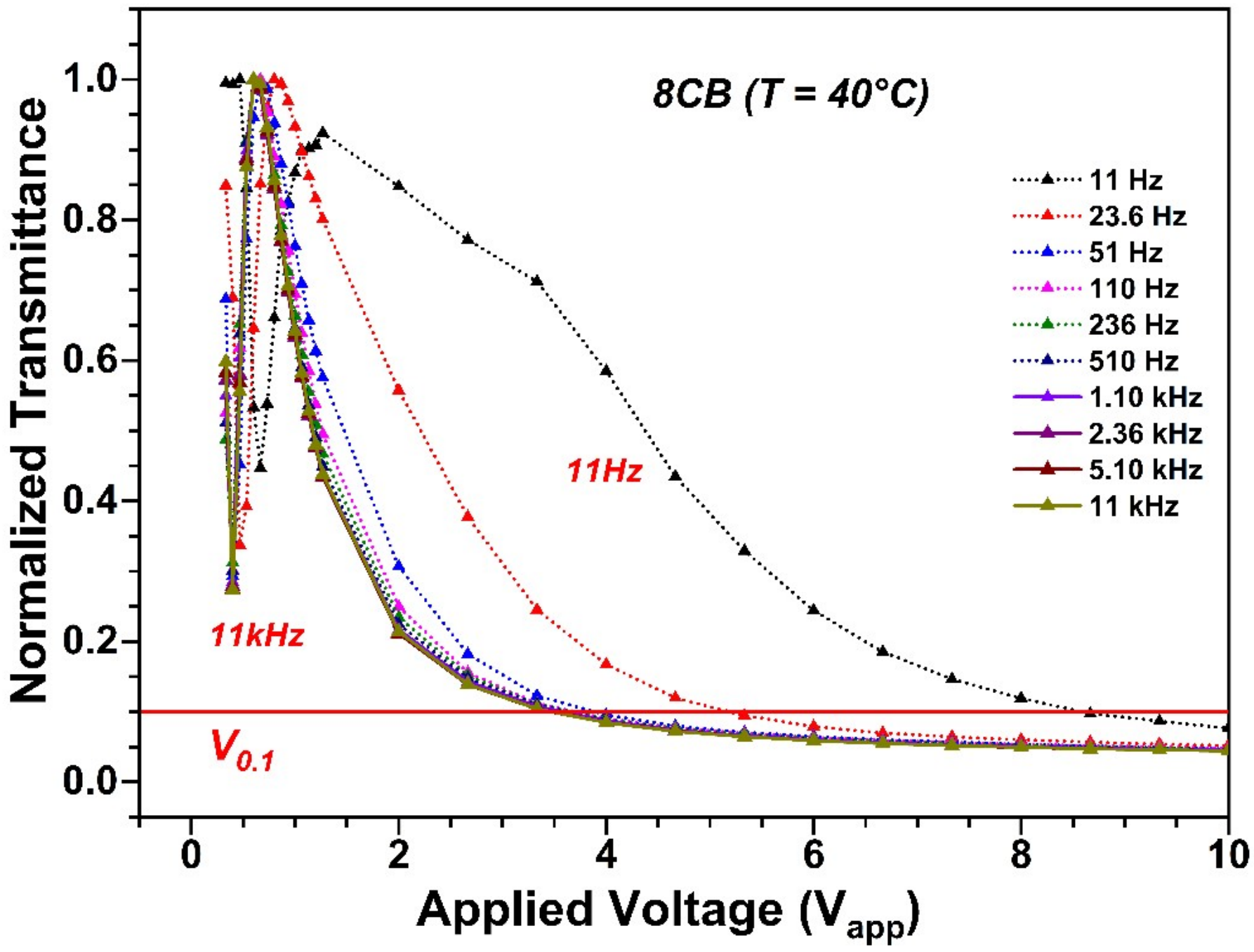

2. Observation of striated texture in the SmA phase in WJ16 and 8OCB

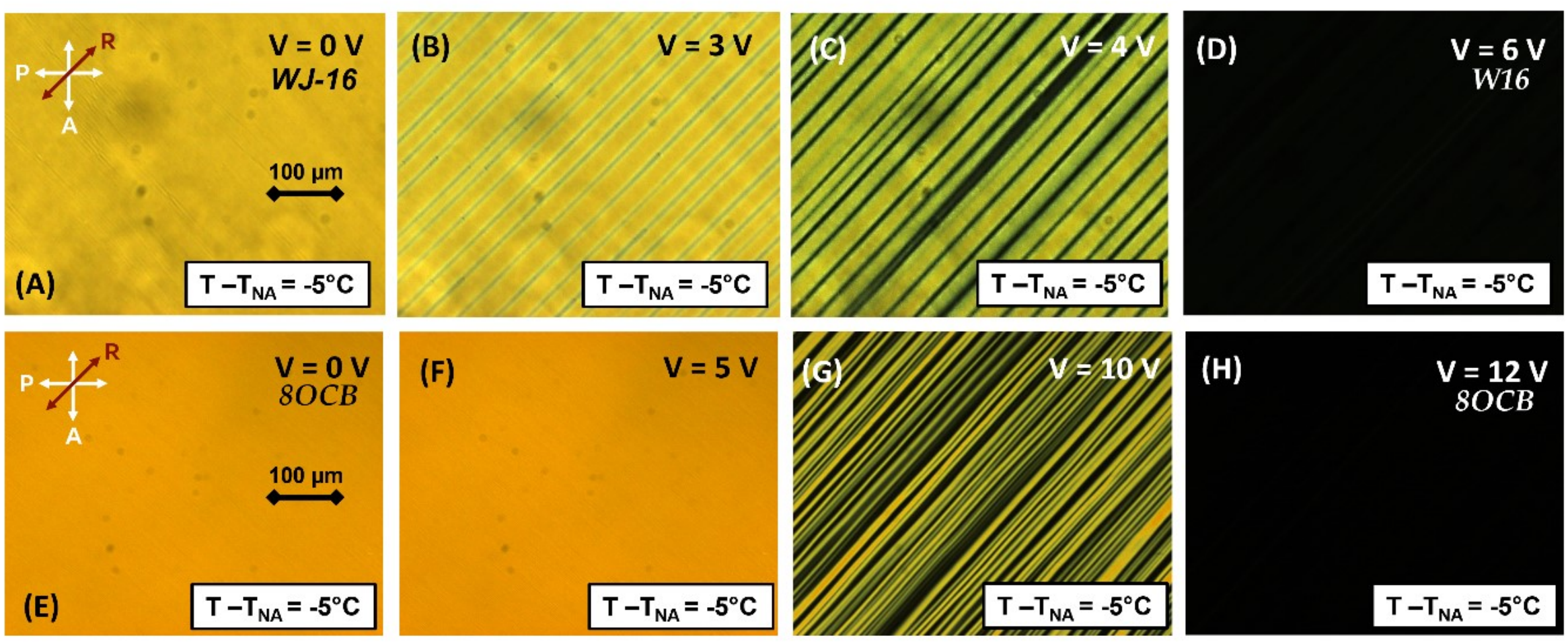

Figure S3: POM textures of a homogeneously aligned planar cell (cell thickness, d = 2µm) filled with WJ-16 (A-D) and 8OCB (E-H) under the effect of applied voltage at 5°C below the NA transition temperature (T_{NA}). The cells are oriented such that the rubbing direction is at 45° to both the polarizer and analyzer. Here A and P refer to the analyzer and the polarizer, while R refers to the rubbing direction. The scale bar shown in 1B and 1F is of length 100 µm and is same for all the textures.

3. Evolution of switching voltage across N - SmA transition in paraelectric LCs:

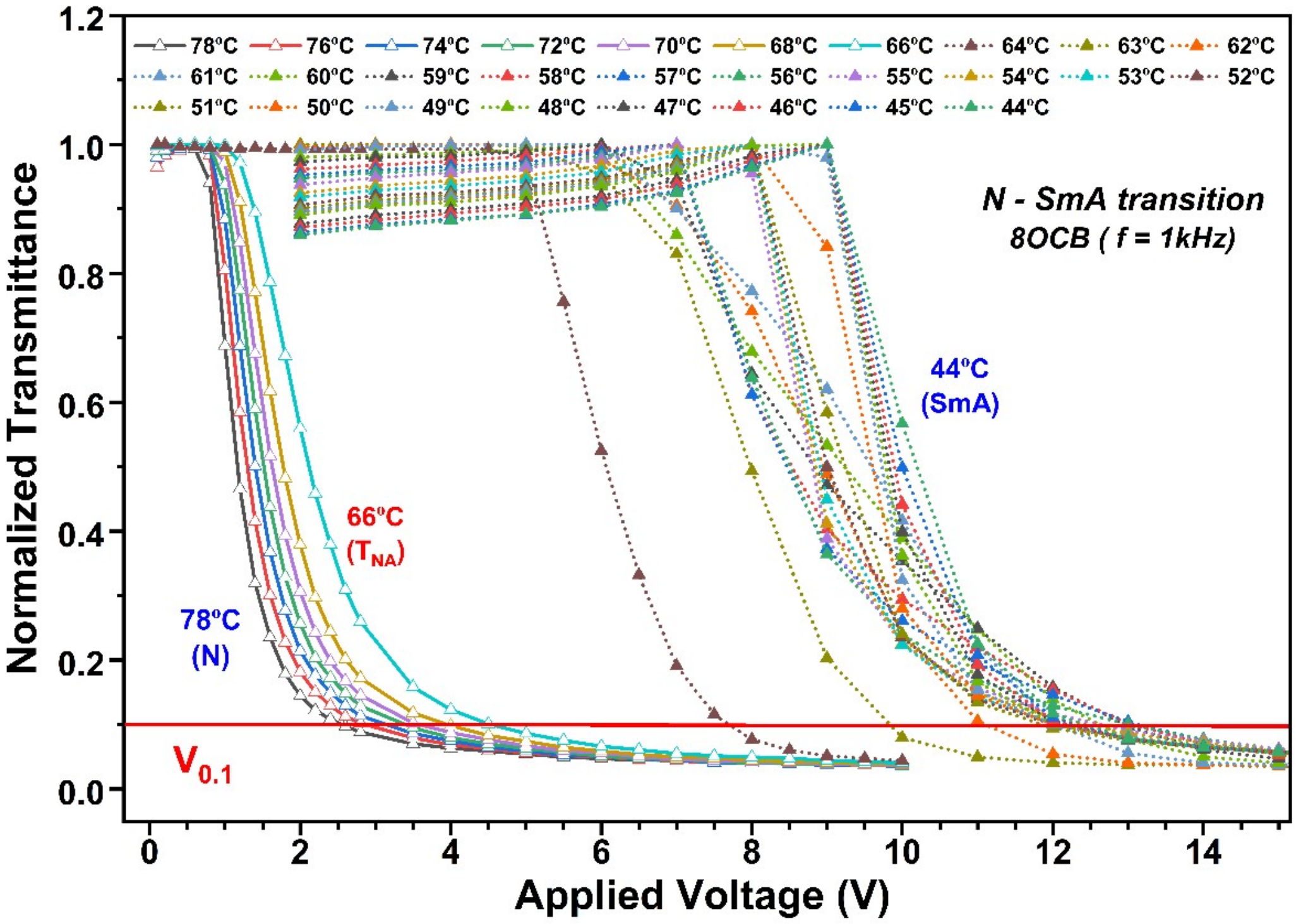

Figure S4: The variation of the normalized transmittance under the application of AC voltage (f = 1kHz, square waveform) for 8OCB as a function of temperature. Here, the solid lines indicate the nematic phase, while the broken lines signify the SmA phase. The N to SmA transition temperature (66°C) is shown as T_{NA}.

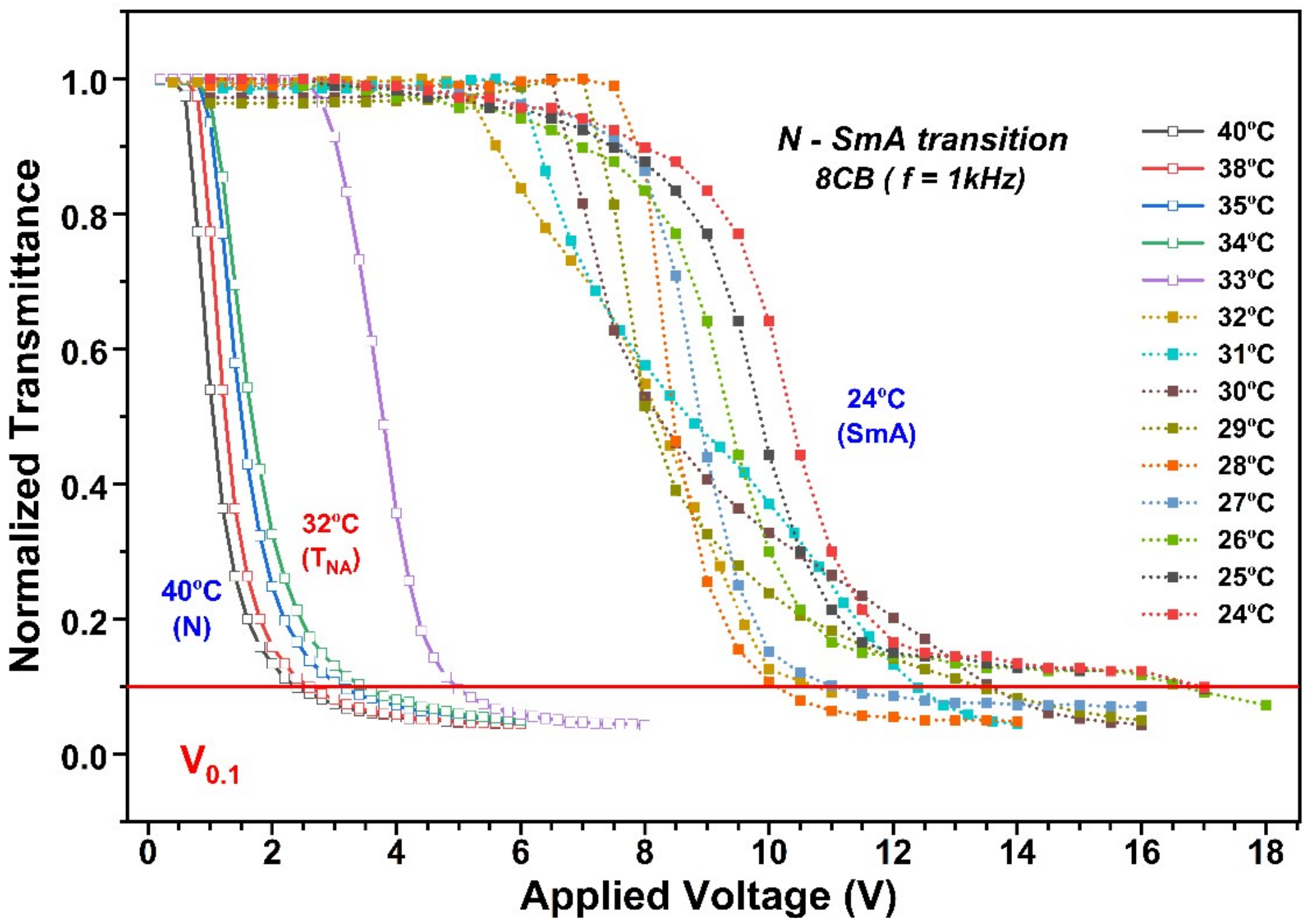

Figure S5: The variation of the normalized transmittance under the application of AC voltage (f = 1kHz, square waveform) for 8CB as a function of temperature. Here, the solid lines indicate the nematic phase, while the broken lines signify the SmA phase. The N to SmA transition temperature (32°C) is shown as T_{NA}.

4. Optical memory effect in WJ-16:

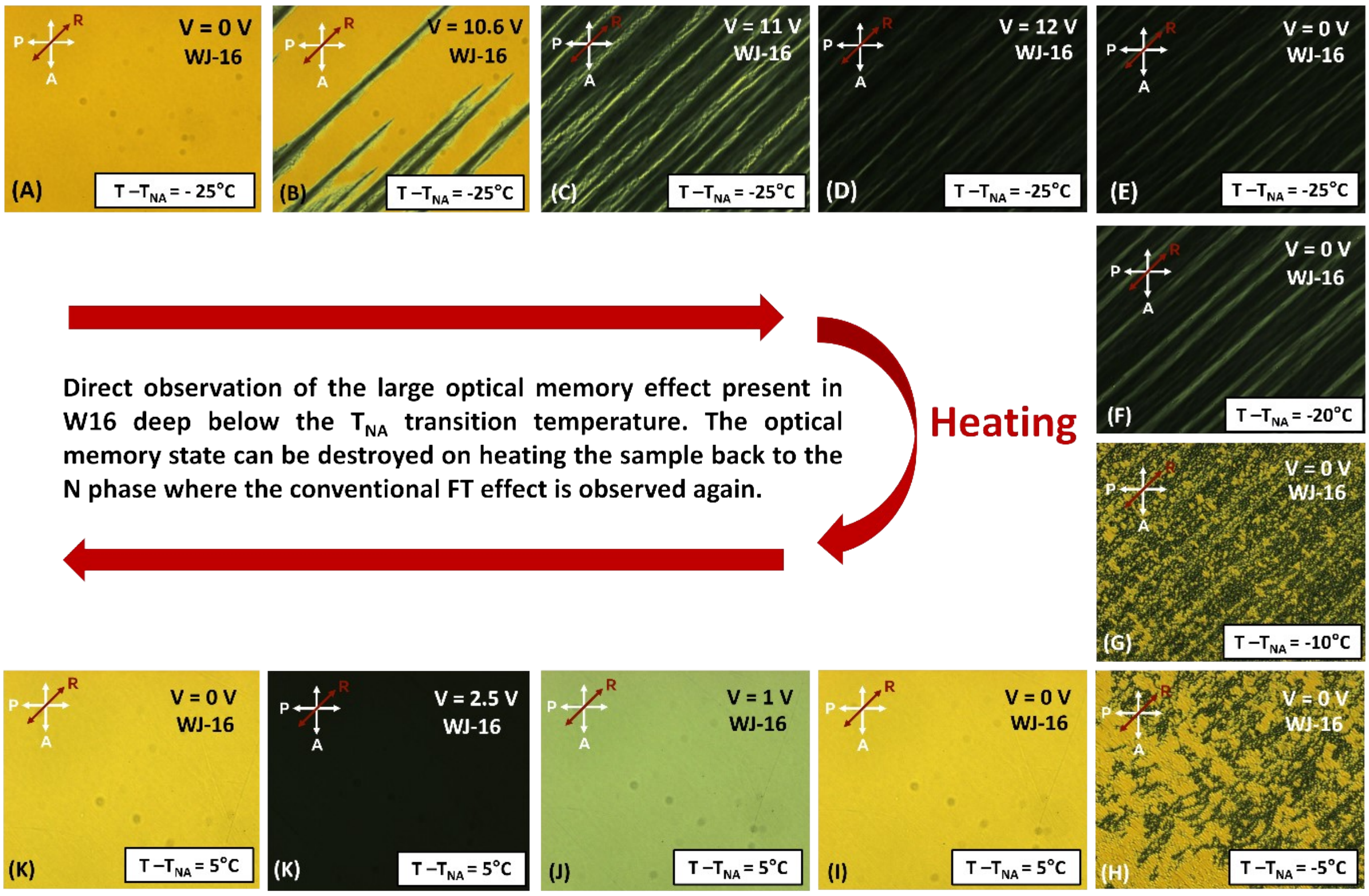

Figure S6: **(A-E)** Optical textures of W16 filled in a planar cell of 2µm thickness showing the existence of complete memory effect at 25°C below the Nematic to Smectic A (T_{NA}) transition temperature. **(F-K)** The memory effect can be destroyed on heating the sample back to its nematic phase effect where the sample shows the conventional Freedericksz transition (FT) effect. The same memory effect can be observed on bringing the molecule back to the SmA phase. This behaviour was tested for a number of cycles and was not observed to deteriorate with repetition.

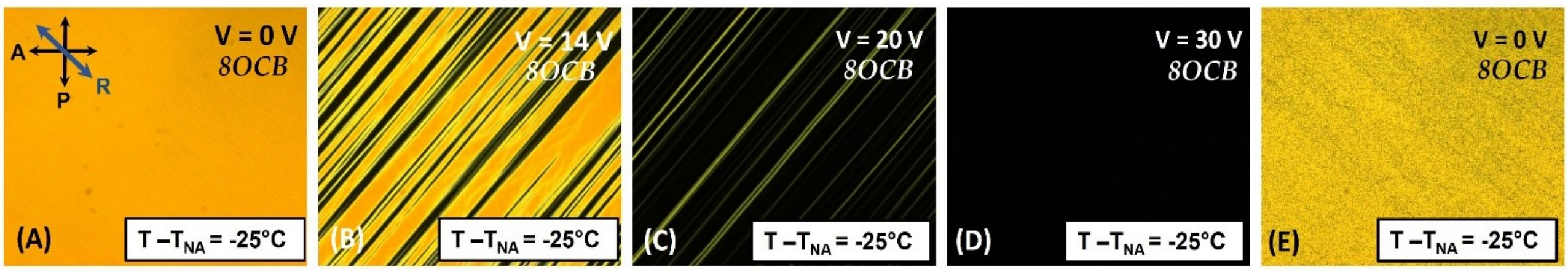

Figure S7: (A-E) POM textures of a homogeneously aligned planar cell (cell thickness, d = 2µm) filled with 8OCB under the effect of applied voltage at 25°C below T_{NA}. The cells are oriented such that the rubbing direction is at 45° to both the polarizer and analyzer. Here A and P refer to the analyzer and the polarizer, while R refers to the rubbing direction.
